# Clinical forensic imaging and fundamental rights in Austria

**DOI:** 10.1080/20961790.2017.1328808

**Published:** 2017-05-23

**Authors:** Sophie Kerbacher, Michael Pfeifer, Bridgette Webb, Reingard Riener-Hofer

**Affiliations:** Ludwig Boltzmann Institute for Clinical Forensic Imaging, Ludwig Boltzmann Gesellschaft, Graz, Austria

**Keywords:** Forensic science, clinical forensic medicine, clinical forensic imaging, forensigraphy, fundamental rights, physical examination, criminal proceedings, ECHR

## Abstract

Clinical forensic imaging encompasses the diverse application of imaging procedures that serve the same purpose: to enable the analysis and investigation of criminal activities and consequences of a crime. All kinds of imaging techniques and their corresponding images can be subsumed under “forensigraphy”, a more comprehensive term for forensic imaging created by the Ludwig Boltzmann Institute for Clinical Forensic Imaging in Graz, Austria. As the word forensigraphy suggests, criminal imaging material should be of use in forensic investigations. Ideally, this can lead to new findings that would not have been revealed without the application of imaging techniques and are moreover admissible as evidence in criminal proceedings. However, the admissibility of evidence can only be facilitated through the implementation of clinical forensic imaging techniques into the forensic routine case work, which requires a precise pre-analysis of the corresponding legal framework. Because taking and displaying internal images of a person's body touches upon various aspects of one's physical and psychological integrity, imaging methods in general and clinical forensic imaging methods especially have a strong impact on and interfere regularly with the fundamental rights of the concerned person. Particularly with regard to a possible medical context, certain legal regulations have to be taken into account. Therefore, this paper examines forensic imaging in the field of radiological forensigraphy, specifically its *in vivo* (i.e. clinical) application. It is designed to enlighten readers as to the great significance of legal barriers that emerge from fundamental rights, as laid down in the European Convention on Human Rights (ECHR), when dealing with clinical forensic imaging. As a result, the legal framework of clinical forensic imaging procedures are comprehensively described, the relevant fundamental rights, especially the right to respect for private and family life, the right to data protection and certain procedural guarantees, are concisely presented to further raise awareness regarding the importance of fundamental rights.

## Introduction

Clinical forensic medicine is becoming increasingly relevant for forensic routine case work. Today's standard for a forensic examination of a living person in cases of domestic violence, strangulation, child maltreatment, traffic related or other incidents is an external examination of the body. Its greatest weakness is that internal findings easily escape the (external) forensic evaluation. A solution is presented by clinically well-established radiological methods such as magnetic resonance imaging (MRI) or computed tomography (CT). Both methods provide an additional and objective basis for forensic examinations [1], with MRI being the preferred method for the assessment of victims of survived violence. However, one must consider that forensic imaging, using the same methods and techniques as for clinical imaging, serves a fundamentally different objective. Whilst the latter is focused on diagnosing and treating diseases, the former is of use for illustrating the sequence of events for criminal proceedings and hence improving legal certainty. Therefore, in the context of forensic imaging, a different appraisal of the images obtained is required, meaning that forensic radiological data cannot be interpreted like data for diagnostic and therapeutic purposes [2]. The different approaches are particularly evident when it comes to minor injuries, which can often occur in connection with incidences of domestic violence. On the one hand, they are usually too small to be clinically significant and require no medical intervention or curative measures by the physician. On the other hand, for criminal proceedings, it is of outmost importance that all traces of violence, whether minor or major, are ascertained and accurately documented [2]. Accordingly, the legal framework concerning clinical forensic imaging has to be analysed to ensure the usability of these forensic findings as evidence in court. A closer description of the legal regulations that need to be considered is given in the section below.

The investigation of the juridical basis for the implementation of forensic imaging methods into routine case work is an important goal of the Ludwig Boltzmann Institute for Clinical Forensic Imaging (LBI CFI) in Graz, Austria [3]. At the LBI CFI, various studies [4] are conducted to establish forensic standards for the application of radiological methods on affected persons of physical and/or sexualized violence. The structure of the interdisciplinary institute of the LBI CFI, which consists of experts in forensic medicine, forensic computer engineering, forensic natural sciences and forensic medical law, provides the perfect environment to further these objectives. Experts from various scientific fields with different research focuses, working together in interdisciplinary cooperation and daily interaction, are not only advantageous, but also essential for scientific progress [5].

Juridical research within the LBI CFI aims to investigate and analyse the legal requirements for clinical forensic examinations. The current legal framework has to be constantly measured on the fundamental rights of the Austrian Constitution, which are interpreted and changed subject to the decisions of the Austrian Supreme Court of Justice [6], and especially that of the Constitutional Court of Austria [7]. Furthermore, the Austrian Constitutional Court's decisions have to be seen in light of the rulings of the European Court of Human Rights (ECtHR), with the implementation of the European Convention on Human Rights (ECHR) into the Austrian Constitution in 1958 and 1964 as a result of a lack of fundamental rights in Austria after the Second World War [8].

Therefore, the aim of the present paper is to provide insight into these topics and to illustrate the corresponding legal and practical problems in this field. The reader is introduced to this work via a discussion of the basic legal framework for clinical forensic imaging under the term forensigraphy (see Chapter “Forensigraphy”). The specific issues in the context of radiological forensigraphy are then reviewed in Chapter “Radiological forensigraphy and legal aspects of its application in Austria”. Finally, the connections between clinical forensic imaging and the fundamental rights laid down in the ECHR are explored in Chapter “Clinical forensic imaging and fundamental rights”. The ECHR, which is equivalent to constitutional law in Austria, provides a broader protection than most of the other fundamental rights included in the Austrian Constitution (cf. Art. 53 ECHR) [8].

## Forensigraphy

Nowadays imaging techniques play an important role in our daily lives. A common mobile phone with a camera can preserve snapshots and is able to make visual as well as acoustic recordings [9]. Applications of imaging procedures can be used for various purposes: photographic material can remind us of something; it can explain and clarify something; and moreover its information can be visually recorded for evidence preservation [10]. Analogue and digital photography can be subsumed under the term imaging. Both techniques represent and portray real objects (e.g. persons, things or animals) by creating photographs [10,11]. Furthermore, situations as well as conditions can be “frozen” and saved for future reference. These new, easily accessible and low-cost possibilities are also important in a forensic context and can be utilized accordingly. Criminal imaging material serves law enforcement purposes and is intended for use within forensic investigations. The term forensigraphy was created as an umbrella term for the use of all kinds of imaging techniques with a forensic connection; it circumscribes all forensic applications of imaging procedures. Imaging material, obtained during the investigation of criminal activities including their analysis, is defined by the specific forensic character of the portrayed thing or situation [10,12–14]. Forensigraphy can therefore be characterized by its interdisciplinary nature, as it finds itself spread across diverse scientific domains. Its subcategories are forensic photography, forensic evidential imaging, LIVE forensic imaging and forensic radiology [12]. Photography is the most common method of choice to visually preserve crime scenes, weapons and suspects or to document injured victims of bodily harm [13]. 3D registration and/or overview photographs help to analyse the timely sequence of events and detailed photographs preserve traces for later analysis [15]. Within clinical forensic medicine, it is important to register the physical state of victims or suspects by taking pictures close to the time of the crime to best preserve evidence later submitted in court [16]. In forensic evidential imaging, various traces are physically and/or chemically characterized  [12]. For example, techniques such as infrared imaging can be applied to determine chemical composition and analyse the distribution of particles in automotive paint samples. Samples can be further compared with a suspect's vehicle to establish connections in traffic accidents [17]. The use of LIVE forensic imaging is helpful for resolving illegal activities or for identifying suspects and/or victims; moreover, the time sequences of events at the crime scene can be reconstructed. Forensic radiology, as an interface between medicine and forensic sciences, focuses on the use of radiological procedures and provides important data that can be used by the public prosecution in criminal proceedings [12]. The term medical imaging, which gives insight into the human body, describes diverse diagnostic imaging methods, characterized by a recording of the internal structure of the body. Forensic radiology is used for the forensic investigation of deceased victims of violence as well as the clinical forensic examination of survived victims of violence. The forensic application of medical imaging procedures to investigate injuries of living persons is quickly developing into an important field in radiology [18].

It is important to emphasize that by the accurate and diligent use of forensigraphy, legal certainty can be strengthened, from which not only the criminal justice system, but most importantly the victim or unjustly accused suspect can benefit [12,14].

## Radiological forensigraphy and legal aspects of its application in Austria

Legal certainty can only be achieved if the applied medical method is legally permissible. Therefore, it is important to consider that medical imaging techniques, whether they are newly developed or have already been established in practice, must adhere to existing legal frameworks. Following an overview on radiological forensigraphy, clinical forensigraphy is defined and the corresponding legal regulations are discussed in detail.

### Basics

As previously discussed (cf. Chapter “Forensigraphy”), forensigraphy deals with imaging material, which is directly or indirectly associated with a criminal matter; hence, the term forensigraphy is used as a comprehensive term for criminal imaging [13]. Radiological forensigraphy relies on medical imaging (e.g. X-rays, CT, MRI or ultrasound) and a specific application of these methods for forensic purposes. These applications deliver imaging data from within human bodies, whether these are victims or suspects, for the investigation and analysis of criminal offences. The scanned objects can be people who are examined by a physician for forensic reasons. Therefore, this part of forensic imaging represents an important interface between forensic science and medicine. In this context, forensic imaging has to consider the legal requirements that arise from medico-legal issues as well as in criminal proceedings [14].

Contrary to commonly held public perceptions, forensic medicine is not exclusively concerned with the examination of deceased persons, but also includes the examination of survived victims of bodily and/or sexual violence or suspects, and the documentation and assessment of their injuries [1]. Under these stipulations, it is important to consider the legal framework for the *in vivo* application of imaging procedures, as discussed in Chapter “Clinical forensigraphy”. Furthermore, in the application and use of imaging for forensic purposes, one should not lose sight of the protection of fundamental rights (described in detail in Chapter “Clinical forensic imaging and fundamental rights”). Forensigraphy concerns the visual preservation of real objects for the purpose of criminal prosecution. This requires interference with the private sphere, and in cases of clinical forensigraphy, possible encroachments of one's physical integrity protected by ECHR Article 8 [19,p.10]. The intensity of this interference can vary, regardless of whether it involves naked full-body photographs, X-ray examinations using ionizing radiation or ionization-free methods such as MRI or ultrasound. Because images also represent personal data from individuals, the application of imaging methods and the images themselves always affect the fundamental right to data protection according to the Austrian Data Protection Act (in German: Datenschutzgesetz 2000; DSG 2000 § 1) [20].

### Clinical forensigraphy

As mentioned above, forensic medicine does not exclusively concern the examination of dead bodies, but also the examination of living persons, be they victims of survived violence or suspects. Clinical forensic imaging deals with the application of imaging procedures that are conventionally used in a clinical context. Clinical forensic imaging does not deal with deceased persons, but is concerned with the examination of survived victims of violence and of suspected offenders. The domain of forensic medicine is gaining more and more significance, which may stem from the increasing sensitivity of the general public to cases of domestic violence, violence against women and children and sexual assault. The application of diverse radiological procedures, especially the use of MRI as a radiological procedure without ionizing radiation, offer great opportunities to investigate injuries and ascertain objective information related to medical findings. Using such methods, the extent and type of force exerted against the victim can be examined. With the support of computerized visualization, findings can be prepared and depicted in a comprehensible manner for the layperson, which is essential for fair criminal proceedings and hence legal certainty [21,22].

The relevant legal regulations can be found in the Austrian Code of Criminal Procedure (in German: Strafprozeßordnung 1975; StPO) under the heading of “physical examination”. StPO § 117 (4) defines “physical examination” as “the visitation of body openings, the drawing of blood samples and any other interference into the physical integrity of individuals”^1.^. The legislature interprets “other interference into the physical integrity of individuals” inter alia as the examination by means of imaging procedures [23, p.173], meaning that examinations using X-rays and CT (although these represent a low health hazard) as well as ultrasound waves and MRI are subject to the legal definition of the “physical examination” [24]. Thus, clinical forensic scans fall under the definition of a “physical examination”, which is regulated by StPO § 123 [25]. A “physical examination” shall only be admissible if, on the basis of certain facts, it can be assumed that

(1) a person has left traces, of which ascertainment and examination are essential for the investigation of a criminal offence (StPO §123 s 1(1));
(2) a person conceals objects in his or her body, which have to be secured as evidence (StPO §123 s 1(2)); or
(3) if facts, which are of decisive importance for the investigation of a criminal offence, cannot be ascertained in a different manner (StPO §123 s 1(3))^2.^.

Section 1 (2) is used when a person is suspected of having swallowed foreign bodies such as drugs (e.g. “body packers”), jewellery or microchips [26,27]. In cases of violence against a person, Section 1(1) is seen as lex specialis to Section 1(3) in that the ascertainment of relevant traces is also interpreted such that examinations are allowed to be performed on a person to assign relevant traces at the crime scene to the examined person. Therefore, not only the collection of body fluids such as blood or sperm but also body scans can be carried out as an “physical examination” according to StPO § 123. However, it is important to take the principle of proportionality (StPO § 5) into account [28] when deciding if an external examination is a lesser encroachment on the physical integrity of the person than a body scan to yield the desired result (cf. Chapter “Relevant procedural guarantees”) [27].

The basic condition for the permissibility of a body scan is an order from the public prosecutor's office on the basis of judicial authorization. In cases of exigent circumstances, judicial authorization can be obtained at a later date. However, if permission is not obtained, the results of the examination have to be destroyed immediately pursuant to StPO § 123 s 3. Section 4 of the same regulation (StPO §123) states that the examination itself generally requires the “informed consent” of the victim or suspect. This means that the concerned person has to be clearly informed in advance about the examination and be aware of the possible risks of the imaging procedure so that he or she takes part in the forensic imaging examination of his or her own free will. Afterwards, a physician is instructed to conduct the physical examination by means of the radiological imaging procedure that he or she considers suitable for the purpose of providing relevant results in accordance with StPO § 123 s 5. The legislator foresees only one exception regarding the rule of informed consent: pursuant to StPO § 123 s 4, only a suspect can be forced without consent (meaning against his or her will) to endure a minor encroachment of his or her physical integrity comparable to the drawing of a blood sample. Furthermore, according to the same section of the StPO, the physical examination has to be necessary for solving a criminal offence threatened with more than five years of imprisonment (e.g. bodily harm with a fatal outcome in accordance with the Austrian Penal Code; in German: Strafgesetzbuch; StGB § 86 [29]) or a crime according to Part 2, Section 10 (criminal offences against sexual integrity and self-determination) of the StGB [25,27]. Therefore, the question arises, can a body scan be interpreted as a minor encroachment of physical integrity? Taking the general prohibition of the usage of ionizing radiation for non-medical purposes within the meaning of the Austrian Radiation Protection Act (in German: Strahlenschutzgesetz; StrSchG § 4 s 3) [30] and the decisions of the Austrian Supreme Court of Justice [31,32] into account, every radiological method using ionizing radiation (e.g. X-ray and CT) is forbidden. Hence, regardless of the practicable feasibility, a suspect could only legally be forced (StPO § 93 [33]) to endure a clinical forensic examination and/or a body scan based on a method free of ionizing radiation (e.g. MRI or ultrasound). Moreover, the legislator foresees in StPO § 123 s 7, that the results of a clinical forensic examination or scan are only admissible in court if all previously mentioned prerequisites are fulfilled. Otherwise, the results cannot be presented as evidence in court.

As the above-mentioned explanations have demonstrated, every physical examination as well as every body scan, regardless of the imaging modality used, interferes with and/or affects an individual's physical integrity, which is protected under ECHR Article 8 [19,p.10]. Additionally, data originating from a forensic imaging procedure, such as a picture or a patient's personal data, affect the fundamental right to data protection pursuant to DSG 2000 § 1 [20]. Finally, in criminal proceedings, certain procedural guarantees for an individual, which originate from ECHR Article 6 [19,p.9], must also be considered.

## Clinical forensic imaging and fundamental rights

Fundamental rights are constitutionally guaranteed rights. Thus, in contrast to federal and provincial laws, fundamental rights are hierarchically superior because of their basis in constitutional law. Under the ECHR (granting universal rights), fundamental rights in Austria ensure that a person, regardless of his or her citizenship, have directly enforceable rights and freedoms against state power. Chapter “Overview on fundamental rights” discusses in detail the concept and meaning of fundamental rights and in doing so serves as an ideal starting point to capture the legal barriers relating to clinical forensic imaging (see Chapter “Clinical forensic imaging in light of fundamental rights”).

### Overview on fundamental rights [34–36]

Fundamental or basic rights are based on the idea that barriers are imposed on the mighty and unrestricted state power for the protection of individuals. Thus, the individual gains liberties via fundamental rights. As these are embedded in the Constitution (“constitutionally guaranteed right” according to the Federal Constitutional Law (in German: Bundes-Verfassungsgesetz; B-VG Article 144 s 1) [37,p.122]), they enjoy greater legal validity according to B-VG Article 44 s 1 [37,p.47]. Furthermore, they have to be legally enforceable by an individual, which is guaranteed in Austria by the institution of the Constitutional Court of Austria [7] and the administrative courts for public law, and by the Austrian Supreme Court of Justice [6] for private law. Each fundamental right circumscribes the liberty it grants via a scope of protection, which (because of the general terms in which fundamental rights are worded) must be interpreted. Nevertheless, a legal act (i.e. law, regulation, administrative act or judgment) is allowed to interfere with the scope of protection or protected area, if the interference is justified. During the examination to justify a legal act, the Constitutional Court of Austria considers whether the act

(1) follows a legitimate aim of public interest;
(2) is suitable to achieve said aim;
(3) poses as the most moderate means to reach said aim; and
(4) if, in terms of proportionality, adequacy is given between said legitimate aim and the interference into the concerned fundamental right.

This examination system is called the principle of proportionality and is also regularly applied by the ECtHR.

Because there is no provision for the incorporation of constitutional law into the main document of the Austrian Constitution, that is, the B-VG [37], there is no uniform list of fundamental rights found in the Constitution. In comparison, for example in Germany, the Basic Law for the Federal Republic of Germany 1949 (in German: Grundgesetz für die Bundesrepublik Deutschland; GG Article 79 s 1) [38,p.65] demands the incorporation of such rights directly into the Constitution. The two most important constitutional catalogues in Austria are the Basic Law on the General Rights of Nationals (in German: Staatsgrundgesetz; StGG) [39] and the ECHR [19]. In addition to these two catalogues, separate provisions, for example, B-VG Article 7 (the principle of equality) [37,p.8] and Article 83 s 2 (the right to a trial before the lawful judge) [37,p.77] as well as separate constitutional laws (so-called “Bundesverfassungsgesetze”), have to be considered as fundamental rights. Examples of separate constitutional laws are the Personal Liberty Act (in German: Bundesverfassungsgesetz über den Schutz der persönlichen Freiheit) [40], the Federal Constitutional Act on the Rights of Children (in German: Bundesverfassungsgesetz über die Rechte von Kindern) [41] and the Rights of Home Act (in German: Gesetz zum Schutze des Hausrechts) [42]. Moreover, individual provisions in state treaties, for example, the freedom of religion in the State Treaty of Saint-Germain-en-Laye (in German: Staatsvertrag von Saint-Germain-en-Laye Article 63 [43,p.13]) or federal laws such as the DSG 2000 § 1 [20] are fundamental rights. By applying the law of the European Union, the Charter of Fundamental Rights of the European Union (CFR) [44] can also be relevant. Furthermore, recent decisions of the Constitutional Court of Austria in connection with B-VG Article 7 (the principle of equality) [37,p.8] recognize the application of the CFR beyond the scope of EU law [45]. Despite the abundance of different sources of fundamental rights in Austria, the ECHR has a prominent position because, on the one hand, the convention (a treaty of international law drafted by the Council of Europe in 1949) was ratified in Austria in 1958 and transformed into constitutional law in 1964 [46]. On the other hand, the ECHR is more often applicable than other fundamental rights because of the wider range of protection it offers (ECHR Article 53 [19,p.27]). Hence, the decisions of the ECtHR play a central role in the interpretation of the ECHR by the Constitutional Court of Austria.

### Clinical forensic imaging in light of fundamental rights

After these short introductory remarks on fundamental rights, the intersecting aspects of clinical forensic imaging (described in Chapter “Radiological forensigraphy and legal aspects of its application in Austria”) and fundamental rights shall be presented. Therefore, it should be noted once again that forensigraphy can interfere with the following fundamental rights: the right to respect for private and family life within the meaning of ECHR Article 8 [19,p.10], the right to data protection pursuant to DSG 2000 § 1 [20] and procedural guarantees derived from ECHR Article 6, the right to a fair trial [19,p.9].

#### The right to respect for private and family life [27,34-36,47]

ECHR Article 8 s 1 states that “[e]veryone has the right to respect for his private and family life, his home and his correspondence” [19,p.10]. According to the legislature and prevailing literature, the right to respect for private and family life grants free development and self-determination over one's physical, mental and spiritual identity [48,49]. This protection is not restricted to the private sphere, but also extended (to a degree) to the public sphere [48,50,51], whereby the range as well as the complete definition is still not conclusively clarified. The self-determination of one's own physical integrity is, however, an important part of the protective scope of ECHR Article 8, which is interfered with by any decision without the consent of the person concerned. In addition, physical integrity is narrowed by interpretation and, therefore, includes both physical and psychological integrity.

With regard to clinical forensic imaging modalities, every act, be it a photograph or a body scan, thus constitutes interference to the right for self-determination over one's body or physical integrity. This would mean that the use of force to obtain a forensic image or perform a scan would be prohibited against anyone, including the suspect of a criminal offence. However, interference into this protected area is permitted “such as is in accordance with the law and is necessary in a democratic society in the interests of national security, public safety or the economic welfare of the country, for the prevention of disorder or crime, for the protection of health or morals, or for the protection of the rights and freedoms of others” pursuant to ECHR Article 8 s 2 [19,p.10]. The “necessity [for interference] in a democratic society” is ensured by the principle of proportionality (see Chapter “Overview on fundamental rights”). Thus, a forensic image, which qualifies as interference in ECHR Article 8 s 1, is legal if it is based on a law (e.g. StPO § 123) and if this law is in accordance with the principle of proportionality (StPO § 5). Therefore, obtaining a forensic image has to be necessary on a case-by-case basis and must additionally be the most moderate process by which results for the clarification of a criminal offence can be acquired. Thus, a distinction is drawn between forensic imaging approaches regarding victims and those related to suspects: a victim can never be forced to endure a forensic scan without “informed consent”. However, in the case of a suspect, the compulsion to undergo an imaging procedure depends on the degree of public interest (here, the offence itself), can be proportional and hence permitted (cf. StPO § 123 s 4 [25]).

#### The right to data protection [34-36]

The legal framework concerning data protection is determined by European law, specifically Directive 95/46/EC of the European Parliament and the Council of 24 October 1995 on the protection of individuals with regard to the processing of personal data and the free movement of such data [52]. Data protection itself is also a part of the right to respect for private and family life and therefore protected under ECHR Article 8.

In Austria, data protection is regulated by the DSG 2000, with § 1 possessing constitutional rank and guaranteeing everyone a “right to secrecy of personal data, insofar as a legitimate interest exists”^3.^ [20]. Personal data, be it automated or manual processed data, contain information about a person, regardless of whether these are private or public data (e.g. data about someone's professional life). The right to secrecy against third parties exists, however, only in respect to secret data. Such data are defined as that which are accessible only to a limited number of people (e.g. data uploaded to Facebook that are not restricted exclusively to Facebook friends are not covered). The legitimacy of secrecy itself is judged on the basis of objective criteria.

The Austrian Data Protection Act (DSG 2000 § 1 s 3 (1) and (2)) grants everyone the right to information, the right to rectification and the right to delete his personal data protected by secrecy. According to DSG 2000 § 1, interference with the right of data protection may only occur if it is in the vital interest of the person concerned or with his consent and serves the purposes mentioned in ECHR Article 8 s 2. Therefore, an obligation binds not only the legislator to provide an appropriate legal basis for personal data protection (here, specifically the DSG 2000), but also the data-processing persons to the duty to secrecy. According to DSG 2000 § 4 (4) and (5) [53], both the “controller” (in this case the public prosecutor's office) and the “processor” (here a physician conducting a clinical forensic examination) are subject to the obligation of secrecy when clinical forensic imaging is undertaken in criminal proceedings.

#### Relevant procedural guarantees [26, 34-36,47]

Procedural guarantees obligate the legislator to implement and formulate fundamental guarantees for civil or criminal procedure by law. In Austria, there are as follows:

(1) the right to liberty and security (also called the right to personal liberty) laid down in the Personal Liberty Act (in German: Bundesverfassungsgesetz über den Schutz der persönlichen Freiheit) [40] and in ECHR Article 5 [19,p.7],
(2) the right to a fair trial according to ECHR Article 6 [19, p.9],
(3) no punishment without law within the meaning of ECHR Article 7 [19, p.10], and
(4) the right to a trial before the lawful judge, which is embedded in B-VG Article 83 s 2 [37, p.77] as well as in ECHR Article 6 [19,p.9].

These fundamental rights, therefore, do not target a specific liberty that must be granted by the state, but are directed at the state and the actions of the administrative and judiciary powers. Nevertheless, the principle of proportionality, which is in general an essential part of the so-called freedom rights or liberties expressed in Section 2 of ECHR Articles 8 to 11 [19,p.10], plays an important role in the legal design of these fundamental rights.

In criminal procedure law, the maxim *nemo tenetur se ipsum accusare* is a vital basic principle, and states that no person can be compelled to accuse him or herself. The ECtHR derives this from the principle of fairness laid down in ECHR Article 6 [54]. In Austria, the Constitutional Court deduces this from the principle that for “criminal proceedings the procedure is by indictment” according to B-VG Article 90 s 2 [37,p.80] and this regulation is in turn based on ECHR Article 6 [55,56]. Thus, a suspect would not be obliged to provide his or her body as evidence during the course of a physical examination using forensic imaging methods. However, taking into consideration permitted interference with the right to respect for private and family life according to ECHR Article 8 s 2 [19,p.10] (see Chapter “The right to respect for private and family life”) and the principle of proportionality, it seems legitimate that the maxim *nemo tenetur se ipsum accusare* is exceeded within very narrow limits to provide for the clarification of certain offences involving an increased public interest (e.g. criminal offences with high penalties). Therefore, the differential treatment of victims and suspects of certain criminal offences within the framework of the physical examination as laid down in StPO § 123 s 4 [25] (cf. Chapter “Clinical forensigraphy”) and in particular within the framework of a clinical forensic scan is, in spite of the maxim *nemo tenetur se ipsum accusare*, justified.

Further relevant procedural guarantees derived from ECHR Article 6 [19,p.9] are the principle of fairness (as mentioned above) and the principle of proportionality itself. While in Austria the former is taken into account by the general principles in criminal proceedings (cf. StPO §§ 1 to 17 [57]), the latter is explicitly mentioned as a general principle in StPO § 5 [28] and can also be derived implicitly from several individual regulations in the Austrian Code of Criminal Procedure. For example, the physical examination of a suspect charged with a minor misdemeanour using radiological methods without his informed consent would clearly be disproportionate when referring to StPO § 123 s 4 [25].

## Conclusion

Under the heading of clinical forensic imaging or clinical forensigraphy, technological possibilities are rapidly evolving and provide new opportunities for the forensic physician to detect relevant traces on and within the body of an examined person. All these efforts are directed at achieving increased legal certainty in judicial proceedings. This paper provides legal insight into the sensitivities surrounding new technological advances. In sensitive areas, such as forensic imaging, almost every act leads to the interference of fundamental rights. Hence, it is essential for physicians and affected persons to have a basic knowledge concerning their rights and obligations. Clinical forensic imaging not only enables scientific development in the field of forensic sciences, but above all serves the end user, the judiciary. Therefore, to further forensigraphy as an important interdisciplinary field of science, an interdisciplinary approach including comprehensive legal expertise cannot just be recommended, but is indispensable. Technical innovations have to be embedded in the given legal framework, which is constituted and protected by fundamental rights. In turn, technical innovations also influence legal development and it is an important task of the legislator to ensure that legal regulations are adapted to cover new scientific and technological developments.

## Notes

1. § 117 StPO. Im Sinne dieses Gesetzes ist 4. “körperliche Untersuchung” die Durchsuchung von Körperöffnungen, die Abnahme einer Blutprobe und jeder andere Eingriff in die körperliche Integrität von Personen.

2. § 123 StPO. (1) Eine körperliche Untersuchung ist zulässig, wenn 1. auf Grund bestimmter Tatsachen anzunehmen ist, dass eine Person Spuren hinterlassen hat, deren Sicherstellung und Untersuchung für die Aufklärung einer Straftat wesentlich sind, 2. auf Grund bestimmter Tatsachen anzunehmen ist, dass eine Person Gegenstände im Körper verbirgt, die der Sicherstellung unterliegen, oder 3. Tatsachen, die für die Aufklärung einer Straftat oder die Beurteilung der Zurechnungsfähigkeit von maßgebender Bedeutung sind, auf andere Weise nicht festgestellt werden können.

3. § 1 DSG 2000. (1) Jedermann hat, insbesondere auch im Hinblick auf die Achtung seines Privat- und Familienlebens, Anspruch auf Geheimhaltung der ihn betreffenden personenbezogenen Daten, soweit ein schutzwürdiges Interesse daran besteht.
